# Regional and developmental characteristics of human embryo mosaicism revealed by single cell sequencing

**DOI:** 10.1371/journal.pgen.1010310

**Published:** 2022-08-08

**Authors:** Yixin Ren, Zhiqiang Yan, Ming Yang, Laura Keller, Xiaohui Zhu, Ying Lian, Qi Liu, Rong Li, Fan Zhai, Yanli Nie, Liying Yan, Gary D. Smith, Jie Qiao

**Affiliations:** 1 Department of Obstetrics and Gynecology, Peking University Third Hospital, Beijing, China; 2 Key Laboratory of Assisted Reproduction, Ministry of Education, Beijing, China; 3 Beijing Key Laboratory of Reproductive Endocrinology and Assisted Reproductive Technology, Beijing, China; 4 Department of Obstetrics and Gynecology, University of Michigan, Ann Arbor, Michigan, United States of America; 5 Reproductive Medical Center, Henan Provincial People’s Hospital, Zhengzhou City, Henan, China; UCSF: University of California San Francisco, UNITED STATES

## Abstract

Chromosomal mosaicism is common throughout human pre- and post-implantation development. However, the incidence and characteristics of mosaicism in human blastocyst remain unclear. Concerns and confusions still exist regarding the interpretation of chromosomal mosaicism on preimplantation genetic testing for aneuploidy (PGT-A) results and embryo development. Here, we aimed to estimate the genetic concordance between trophectoderm (TE), inner cell mass (ICM) and the corresponding human embryonic stem cells (hESCs), and to explore the characteristics of mosaicism in human blastocyst and hESCs on a single cell level. The single cell sequencing results of TE cells indicated that 65.71% of the blastocysts were mosaic (23 in 35 embryos), while the ICM sequencing results suggested that 60.00% of the blastocysts were mosaic (9 in 15 embryos). The incidence of mosaicism for the corresponding hESCs was 33.33% (2 in 6 embryos). No significant difference was observed between the mosaic rate of TE and that of ICM. However, the mosaic rate of the corresponding hESCs was significantly lower than that of TE and ICM cells, suggesting that the incidence of mosaicism may decline during embryonic development. Upon single cell sequencing, we found several “complementary” copy number variations (CNVs) that were usually not revealed in clinical PGT-A which used multi-cell DNA sequencing (or array analysis). This indicates the potential diagnostic risk of PGT-A based multi-cell analysis routinely in clinical practice. This study provided new insights into the characteristics, and considerable influences, of mosaicism on human embryo development, as well as the clinical risks of PGT-A based on multi-cell biopsies and bulk DNA assays.

## Author summary

Chromosomal mosaicism is a common biological phenomenon during human embryo development, which may have interferences with clinical PGT-A decision-making. In this study, single cell DNA sequencing and copy number variation (CNV) analysis were performed to estimate the genetic concordance of TE, ICM, and hESCs. The single cell sequencing results of TE cells indicated that 65.71% of the blastocysts were mosaic, while the ICM sequencing result suggested that 60.00% of the blastocysts were mosaic in the 39 embryos we analyzed. The mosaicism may be caused by both whole and segmental abnormalities of the chromosome. Our study described the characteristics of chromosome mosaicism on single cell level in human embryo and brought evidence that mosaicism could raise challenges in the clinical management of PGT-A.

## Introduction

Mosaicism is the presence of two or more cell lines with differing karyotypes within a single organism. Mosaicism is common at human pre- and post-implantation embryo stages [1], as well as in adults’ normal tissues [2]. Chromosomal mosaicisms originate from mitotic errors such as non-disjunction, anaphase lagging, endoreplication, and uniparental disomy during embryonic development [1]. Although there are reports that the transfer of mosaic human embryos yields live births, the mosaic embryo implantation and miscarriage rates are lower and higher, respectively, compared to non-mosaic embryos [3–5]. Clinical usage of preimplantation genetic testing for aneuploidy (PGT-A) has grown considerably over the last decade, with the goal of avoiding transfer of aneuploid embryos, improving implantation rates, reducing miscarriage rates, and improving *in vitro* fertilization (IVF) outcomes and take-home baby rates. Generally, an estimated 5–10 trophectoderm (TE) cells (that develop into the future placenta) are biopsied at the blastocyst stage for genetic testing, with the assumed representation of the chromosome ploidy of the inner cell mass (ICM; that develops into the future fetus) [6]. However, even after PGT-A and euploid embryo transfer, the rate of clinical pregnancy is still relatively low, at about 30% [7]. Several factors can lead to the misdiagnosis of PGT-A, including the physical cellular/DNA damage during embryo biopsy, methodological or technological deficiency of DNA analysis and biological factors [8,9]. Mosaicism is one of the pivotal biological factors, which has raised great concern in recent years for its possible interference in interpretation and clinical decision-making based on PGT-A results [10].

The incidence of mosaicism in human embryos varies with developmental stages and the molecular cytogenetic method used for detection. Previous studies used a cluster of multiple cells from biopsy for array-based comparative genomic hybridization (aCGH) or next-generation sequencing (NGS) to estimate the mosaic rate [11]. Compared with aCGH, which can detect mosaicism when at least 40%-50% of cells with divergent chromosome ploidy are present in the TE biopsy, NGS can identify lower mosaic rates (≥20%) [12]. However, most of previous studies used multi-cell cluster/biopsy for NGS, which may not reflect the real mosaic rate, for chromosome gain and loss might be complementary within a multi-cell cluster/biopsy, yielding an aberrant or missed diagnosis with multi-cell DNA assays. Single cell sequencing, on the other hand, provides an accurate approach for revealing the true relationships between different linages and for resolving cell-to-cell variations [13,14]. Single cell sequencing has been used in the exploration of prevalence of CNVs in somatic tissues and can yield genetic information on a megabase-scale [2,15]. However, regional and developmental genetic concordance in human blastocysts is still unknown.

In this study, we performed single cell sequencing on the TE, ICM as well as ICM-derived hESCs of 39 human blastocysts, and systematically analyzed the spatial and temporal characteristics of CNVs. This study dissected the characteristics, and considerable influences of human embryo mosaicism, and provided a theoretical basis on the interpretation of clinical PGT-A results.

## Results

### Single cell DNA sequencing of human blastocysts

**Fig 1:**
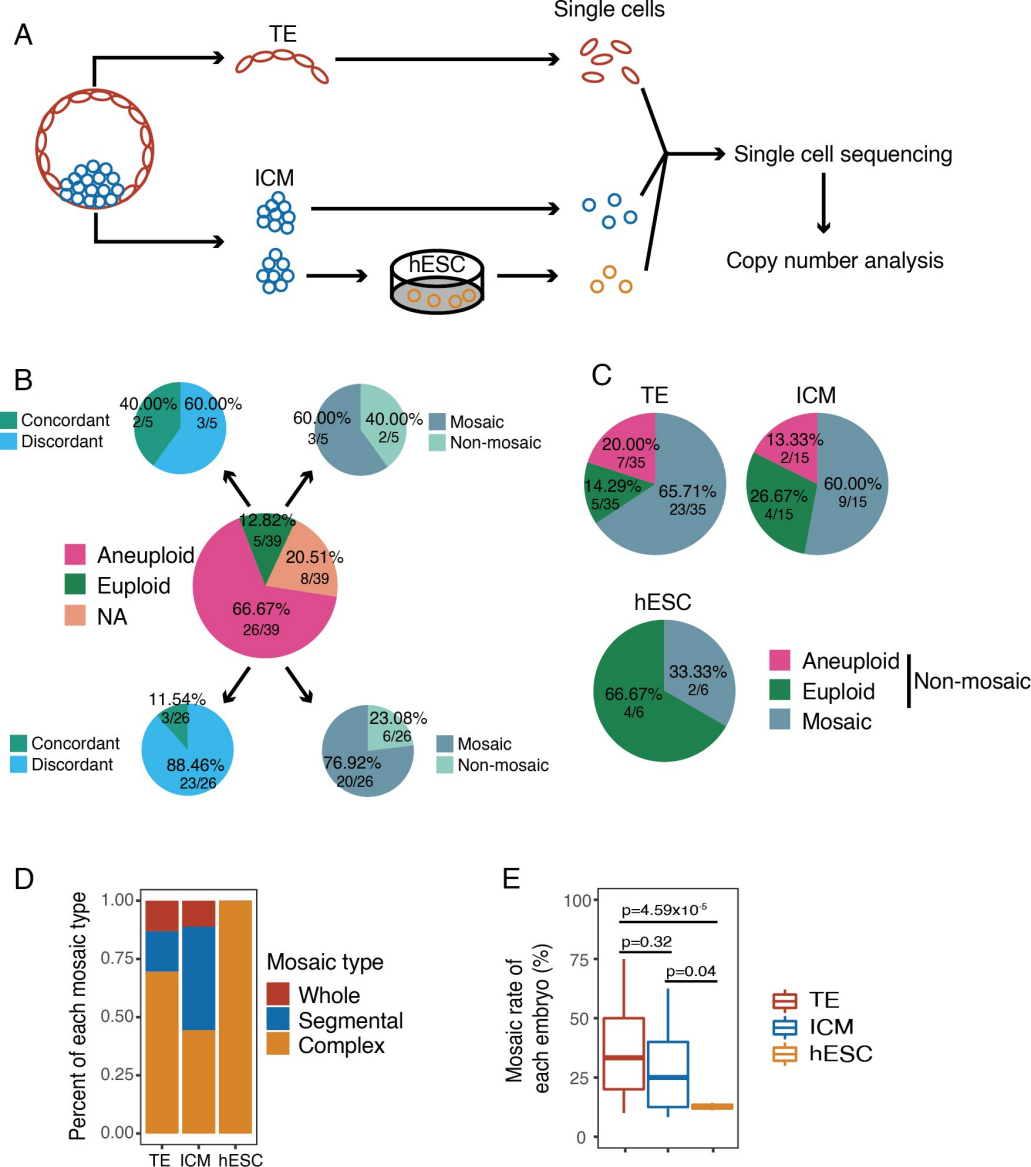
Analysis of chromosome copy number of human embryos. A) Experimental design of the current study. The ICM and TE cells were obtained by biopsy from the donated embryos (n = 39). For ICM cells, some of them were cultured *in vitro* to obtain hESCs. The TE, ICM and hESC were separated into single cells and sequenced. n, number of embryos. B) Pie charts showing the comparison between the single cell sequencing results and the initial PGT-A (multi-cell) results. The center pie showing the summary of the initial PGT-A results based on multi-cell analysis. The top left pie showing the percentage of karyotype concordant and discordant embryos from the initial PGT-A euploid embryos (single cell results vs. initial multi-cell results). If there were one or more cells showing different karyotype with the initial diagnostic results, the results would be identified as discordant. The bottom left pie showing percentage of karyotype concordant and discordant embryos from the initial PGT-A aneuploid embryos (single cell results vs. initial multi-cell results). The top right pie showing the percentage of mosaic and non-mosaic embryos detected in single cell sequencing from the initial PGT-A euploid embryos. The bottom right pie showing the percentage of mosaic and non-mosaic embryos detected in single cell sequencing from the initial PGT-A aneuploid embryos. C) Pie charts showing the percentage of aneuploid, euploid and mosaic embryos in TE, ICM and hESC. D) Rates of different types of mosaicism in mosaic TE (n = 23), ICM (n = 9) and hESC (n = 2). Whole: whole chromosome mosaic. Segmental: segmental chromosome mosaic. Complex: whole chromosome and segmental chromosome mosaic. n, number of embryos. E) Mosaic rates of mosaic TE (n = 23), ICM (n = 9) and hESC (n = 2) of each embryo. Only the mosaic TE, ICM and hESC were shown here. n, number of embryos.

In total, we utilized 39 consented- and donated-blastocysts for this study. The blastocysts were divided into TE and ICM clusters, digested to single cell, and the single cell whole genomes were amplified using multiple annealing and looping-based amplification cycles (MALBAC), following NGS and CNV analysis (Fig 1A). Additionally, we derived hESCs from blastocyst ICMs which were also digested into single cells and sequenced (S1 Fig). This yielded the ability to study aneuploidy and mosaicism within blastocyst regions, between blastocyst regions, and during development to hESC.

On average 1 Gb bases were sequenced in each single cell, corresponding to approximately 0.3× depth (S1 Table). Mapped reads were counted in 1 Mb windows to determine the copy number of each cell by a Hidden Morkov Model (HMM) [16]. To avoid the impacts of physical damage brought from biopsy or amplification bias, data variability was calculated for each sequenced cell and cells with high variable mapped reads were excluded [2]. For each cell, the standard deviation (SD) of the corrected reads number of each chromosome were evaluated and the average of the SD of the top five variable chromosomes was calculated as the variability score (VS) for the cell. To guarantee the accuracy of CNV determination, cells with the top 15% highest VS were excluded (S2A and S2B Fig). The mean VS of retained analyzed cells was 0.34 (S2C and S2D Fig). And there was no significant difference between the VSs of analyzed TE, ICM and hESC (S2E Fig).

In this study, the CNVs of 438 single cells including 285 TE cells, 105 ICM cells and 48 hESCs (S2F Fig) were analyzed and obtained from TE clumps from 35 embryos, ICM clumps from 15 embryos and hESC clumps from 6 embryos (Figs 1C and S2G).

### The incidence of mosaicism in human embryo

Only embryos that were able to provide at least three single cells were used to evaluate the incidence of mosaicism. In this study, we analyzed single cells from 39 embryos, and 31 of them had initial diagnostic karyotypes based on multi-cell analysis. Among the 31 embryos, five embryos were initially diagnosed as euploid and 26 embryos were aneuploid. We compared the results of initial multi-cell diagnosis and those of single cell sequencing. In three of the five initially diagnosed euploid embryos and 23 of the 26 initially aneuploid embryos, the results of single cell sequencing and initially multi-cell analysis were discordant (Fig 1B). Three of the five initially diagnosed euploid embryos, as well as 20 of the 26 initially diagnosed aneuploid embryos, were identified as mosaic embryos by single cell sequencing (Fig 1B and S2–S5 Tables). In TE, the incidence of mosaicism was 65.71% (23/35 embryos), while ICM showed an incidence of 60.00% (9/15 embryos) (Fig 1C). There was no significant difference between the incidence of mosaicism in the TE and ICM (p = 0.70). In hESCs, the incidence was 33.33% (2/6 embryos), which was numerically, yet not significantly, lower than the TE (p = 0.13) and ICM (p = 0.27). Mosaic embryos were classified into three types by the CNV abnormality readout, whole mosaic (whole chromosome mosaic), segmental mosaic (segmental chromosome mosaic) and complex mosaic (both whole and segmental chromosome mosaic). 69.57% (16/23) of embryos were complex mosaic in TE cells, while in ICM cells, the incidences of complex and segmental mosaicism were the same at 44.44% (4/9). In hESCs, the two mosaic embryos were both grouped as complex (Fig 1D). To evaluate the mosaicism level in different cell types, the cell-specific mosaic rates (percentage of cells with discordant chromosome CNV in each individual embryo) were compared within TE, ICM and hESCs. No significant differences were found on the mosaic rates of TE and ICM (37.12% v.s. 28.52%, p>0.05) (Fig 1E). The mosaic rate of hESCs was significantly lower than that of the blastocyst cells (12.70% v.s. 34.26%, p<0.05), suggesting the decreased incidence of mosaicism during hESC derivation and/or expansion. To explore whether the mosaic rates were influenced by the sequenced cell numbers in each embryo, we analyzed the relation between mosaic rates and sequenced cell numbers (S3A Fig). In TE, ICM and hESCs, we did not find an obvious relation between the mosaic rate and the sequenced cell number.

The distribution of whole and segmental abnormalities was analyzed on each chromosome. In TE cells, aneuploidies were more likely to occur on chromosome 19, 21, 22 and X, while segmental abnormalities tended to exist on chromosome 1, 2 and 8. In ICM cells, chromosome 14 and 22 monosomy, chromosome 21 trisomy, and segmental duplication of chromosome 11 were more frequently observed (S3B Fig).

Aneuploidies in embryos were caused by meiotic and/or mitotic errors. In order to know whether mitotic errors were related to meiotic errors, we tracked the origin of CNVs in aneuploid blastocysts (Table 1). Four embryos showed meiotic errors only, which were not mosaic embryos (S2 Table); 19 embryos showed mitotic errors only, which were mosaic embryos (S3 Table); 11 embryos had both meiotic and mitotic errors (S4 Table); five embryos were euploid (S5 Table). Mosaic rates were compared between embryos with and without meiotic errors. No significant differences were observed between the two groups (p = 0.82). This indicated that meiotic errors do not contribute to mosaicism (S4 Fig).

**Table 1:** Aneuploid types of blastocysts.

Meiotic	Mitotic	Meiotic & mitotic
11.76%	55.88%	32.35%

### Mosaicism in the TE, ICM and hESCs of the same embryo

**Fig 2:**
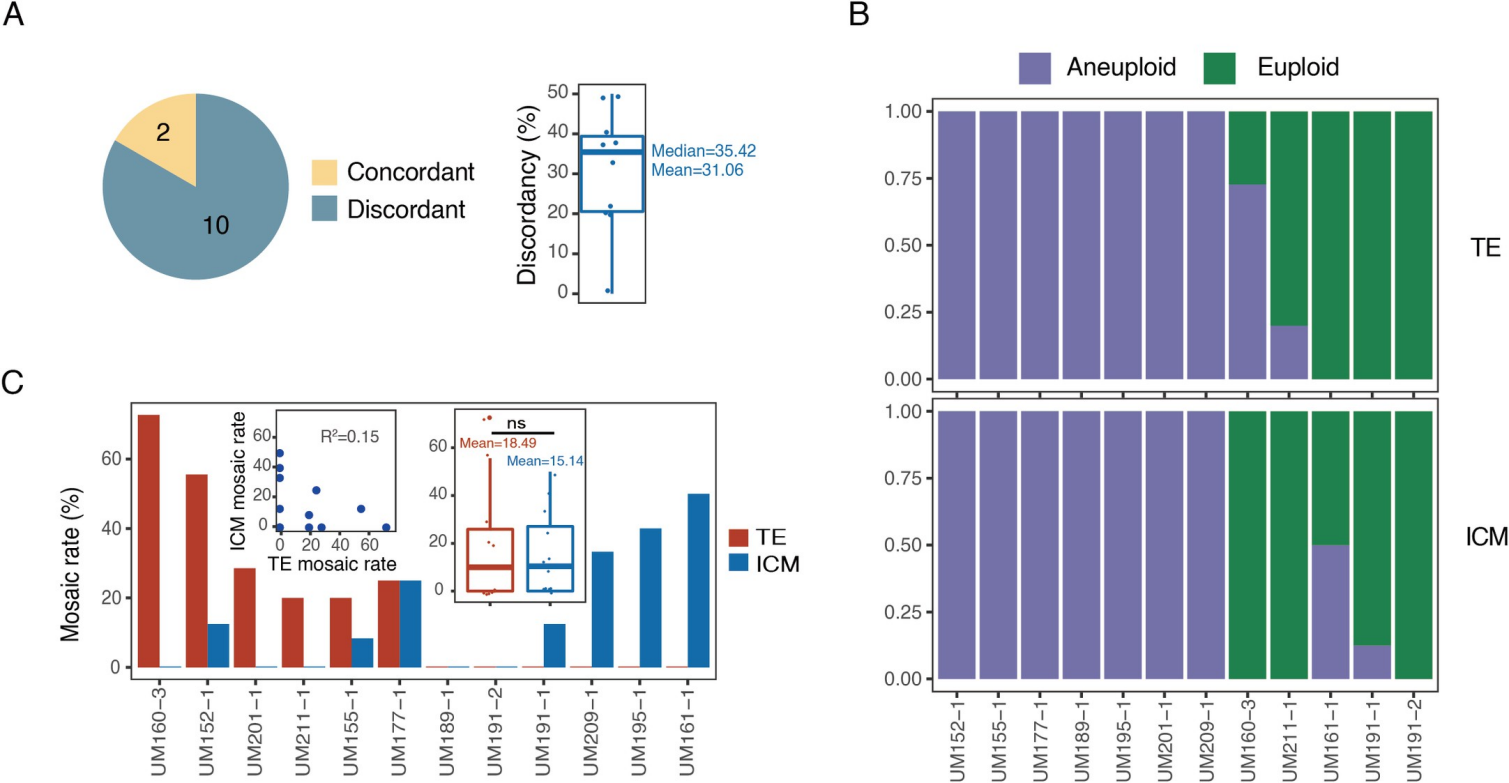
Analysis of chromosome copy number of TE and corresponding ICM from the same embryo. A) Number of TE-ICM concordant (n = 2) and TE-ICM discordant (n = 10) embryos (left) and the discordancy of the 10 TE-ICM discordant embryos (right). Among the 12 embryos having both TE and ICM cells analyzed, 2 embryos showed the concordant chromosome status and 10 embryos showed discordant chromosome status. Of the 10 TE-ICM discordant embryos, they showed a mean of 31.06% discordancy. n, number of embryos. B) Chromosome status of the matched TE-ICM in each embryo (n = 12). Two (UM160-3, UM211-1) of the 12 embryos had aneuploid TE cells but showed euploid ICM cells. Another two (UM161-1, UM191-1) embryos showed euploid TE cells but had aneuploidy ICM cells. n, number of embryos. C) Mosaic rates of the matched TE-ICM in each embryo (n = 12). The barplot showed the mosaic rates of TE and ICM from 12 embryos with both TE and ICM cells. The left insert dotplot showed the relation between the mosaic rate of TE and matched ICM. The right insert boxplot showed the mosaic rates of TE and matched ICM. n, number of embryos.

In this study, 12 embryos yielded both TE and ICM single cell isolation and analysis. CNV analysis showed that 10 of the 12 embryos (83.33%) had differing karyotypes between the TE and ICM (Fig 2A). To further study the degree of TE-ICM difference in each mosaic embryo, we computed the TE-ICM discordance. For each embryo, we used an equal number of cells from TE and ICM and computed the percentage of unmatched karyotype between TE and ICM cells. As shown in Fig 2A, TE and ICM of the same embryo had a mean discordance of 31.06%, which means that when we randomly selected same numbers of cells from TE and ICM in mosaic embryos, 31.6% TE-ICM pairs had different karyotypes. When aneuploidy of TE and ICM in each embryo was compared, two embryos showed euploidy in TE but some of the cells were aneuploid in ICM (UM161-1, UM191-1), and two embryos had aneuploid cells in TE while showing euploidy in the ICM (UM160-3, UM211-1) (Fig 2B). Among the 10 mosaic embryos, three were TE mosaic (mosaicism only existed in TE), four were ICM mosaic (mosaicism only existed in ICM), and the rest were total mosaic (mosaicism existed in both TE and ICM). In order to know whether the mitotic errors had similar tendencies in the TE and ICM, mosaic rates were compared in these 10 embryos (Fig 2C). Five embryos had higher mosaic rates in the TE than that of ICM, while in four embryos, ICM showed higher mosaic rates than TE. There were no significant correlations between the mosaic rates of TE and ICM in each embryo, suggesting that the mosaic rates in TE and ICM within a single embryo were independent.

**Fig 3:**
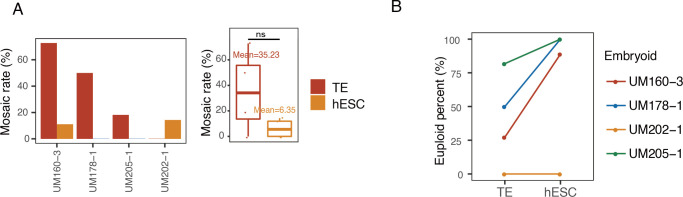
Analysis of chromosome copy number of TE and corresponding hESC from the same embryo. A) Mosaic rates of the matched TE-hESC of each embryo (left) and the corresponding distribution (right) (n = 4). hESC showed lower mosaic rate than corresponding TE in each embryo. n, number of embryos. B) Euploid percent of the matched TE-hESC of each embryo. hESC showed higher euploid percentage than corresponding TE in each embryo.

Mosaic rates were further investigated in TE and hESC from the same embryo. A total of four embryos were collected for this analysis. Although in these four embryos there was no significant difference, the mosaic rate of TE was higher than that of hESCs (Fig 3A). In addition, in those embryos, hESCs tended to have higher euploid rates than the corresponding TE cells (Fig 3B). It is important to note that hESC single cell isolation and analysis were performed following derivation and expansion, thus while hESC were derived from a limited number of ICM, these differences in mosaicism rates may reflect a tendency of mosaicism to decrease during development.

In two embryos both ICM and hESC single cell samples were obtained and evaluated (S5 Fig). The hESCs of UM160-3 and the ICM of UM150-4 were found to be aneuploid. In addition, the mosaic rate in the ICM of UM150-4 was as high as 60%, while the mosaic rate in the hESCs of UM160-3 was relatively lower at 11.11%.

### Single cell sequencing uncovered aneuploid cells from “euploid” cell clumps

**Fig 4:**
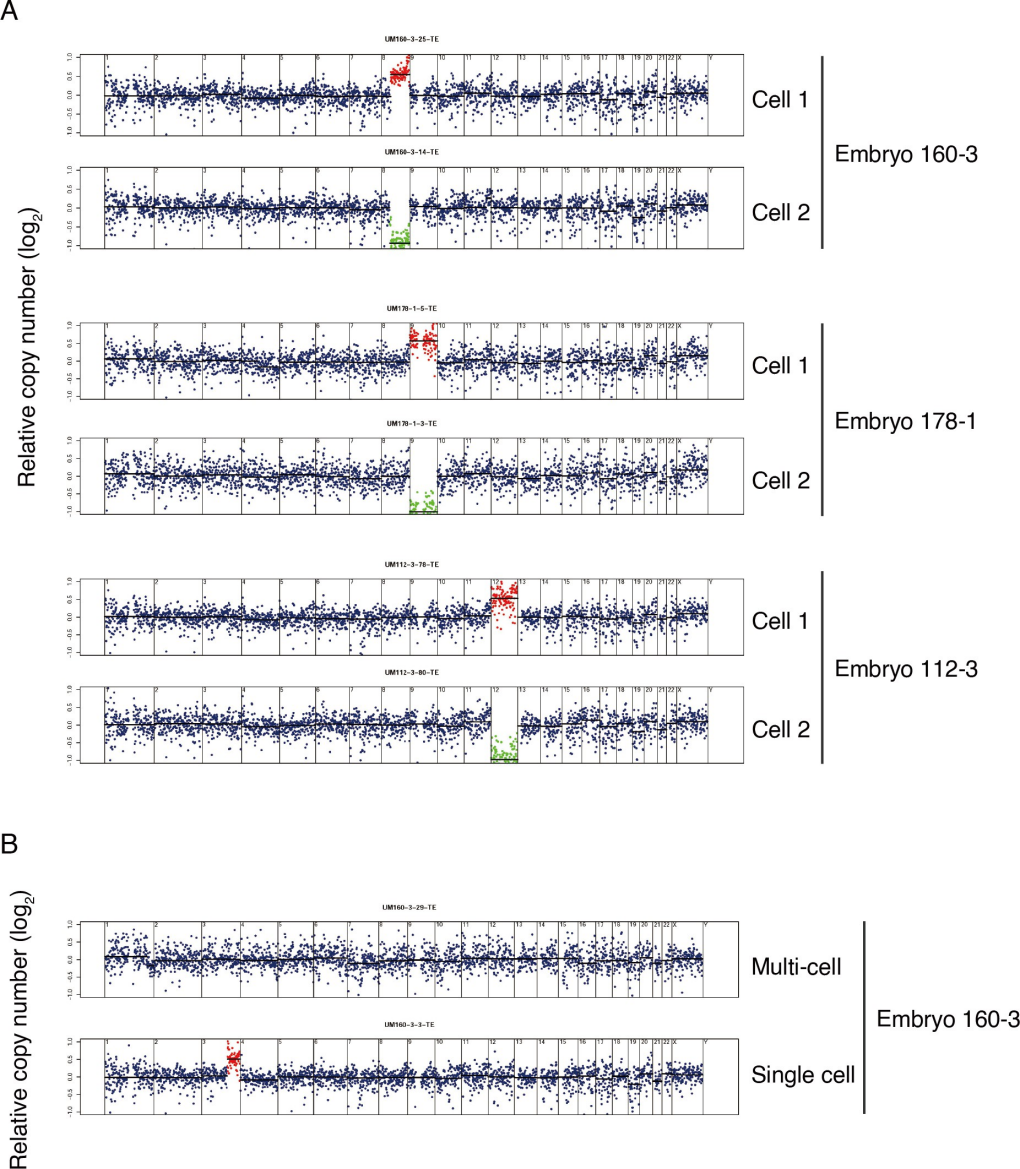
Single cell whole genome sequencing identified chromosome gain and loss which is missed in multi-cell TE sequencing. A) Segmentation plots showing matched chromosome gain and loss at single cell level. Segmentation plots showing copy number of single cells relative to a euploid reference on a log2 scale. Segments above threshold for gain are colored in red, segments below threshold for loss are colored in green. B) Segmentation plots showing chromosome copy number of multi-cell (top) and corresponding single cells (bottom). The multi-cell sample showed euploidy but the corresponding single cell showed a segmental chromosome gain.

In clinical PGT-A, blastocyst TEs are biopsied with 5–10 cells (an estimated number of cells; cell clump) removed, with collective DNA preparation, and analysis for CNVs. Therefore, clinical PGT-A diagnosed euploid results based on a TE cell clump may not represent a euploid state of cells at a single cell level. In this study, by comparing ploidy patterns between different individual cells within each embryo, CNV "complementation" in two individual cells originating from one cell due to mitotic errors was detected (Fig 4). Among the complement cells, one pair had segmental chromosomal aberration, and the other two had whole chromosomal aberration (Fig 4A). When evaluating both single cell and cell cluster genetics, further discordance was observed. For example, in UM160-3, the cell cluster sample was euploid (46, XX), while the corresponding single cell analysis was aneuploid (Fig 4B). This indicated that the aneuploidy could be covered by cluster/bulk DNA assays, which might cause false-negative results in PGT-A.

## Discussion

Mosaicism was firstly recognized in chromosomal disorders several decades ago [17,18]. Its possible impact on early human embryo development and clinical PGT-A result interpretations has generated concern and confusion during the last decade. Many investigators have reported the incidence of early embryo mosaicism, with a wide range from 2% to about 70% [9,19,20]. All of the studies were based on PGT-A results generated from multi-cell biopsies performed on preimplantation embryos in clinical settings. And recently it was reported that single cell RNA sequencing (scRNA-seq) could be used to reveal the pattern of aneuploidy and mosaicism [20]. However, the mosaicism patterns in human embryos still require detailed evaluation by single cell DNA sequencing with contemporary NGS, a sequencing platform currently being used in clinical PGT-A.

In this study, the incidence of mosaicism was found to be relatively higher than many researches based on multi-cell TE biopsy [12,21], which might due to DNA analysis methodological differences. First, compared with fluorescence *in situ* hybridization (FISH), aCGH or PCR-based methods, NGS can detect minor genomic changes with higher sensitivity [11,22]. Second, in past studies, multi-TE biopsied cell masses were used to evaluate the aneuploidy pattern, which would ignore the un-biopsied cells. Furthermore, as most clinical TE biopsies assumed removal and assessment of 5–10 cells, the actual cell number was unknown, and the mosaicism determination was an indirect read-out based on CNV graphical representation. In our study, if there was even only one cell whose chromosomal copy number was divergent from others detected, the embryo would be identified as a mosaic embryo. We carried out several strategies to guarantee the accuracy of the reported CNV and determine the reliable CNV in each single cell (see Method section). Thus, the mosaicism incidence we detected was more similar to other estimated and measured single-cell researches [20,23,24].

We further compared the characteristics of aneuploidy between TE and ICM, as well as blastocyst cells and hESCs. Because hESCs collected from one individual clone tend to have the same ploidy, we picked cells from different clones to minimize this bias. In this study, we found the mosaic rate in hESCs was significantly lower than that of TE and ICM in the samples detected. In addition, in the same embryo, although there was no significant difference, the mosaic rate of hESCs were lower than that of the TE cells, indicating that mosaicism declined with development to some extent. However, the sample size in our study was limited, more samples are required to validate this conclusion. A study reported that in 43 previously diagnosed mosaic embryos, 28% of embryos had normal ICMs with normal TE specimens, which indicated aneuploid cell lines could only be distributed in limited areas of the TE [25]. Furthermore, if there was even one cell showing a different karyotype from the initial multi-cell diagnostic result, the results of single cell sequencing and initially multi-cell analysis would be identified as discordant. This could also explain the discordance between the clinical diagnosis results and our single cell sequencing results. In addition, the limitation of cell numbers of each embryo could also lead to bias. Because of the rarity of human embryos and the technique limitation of laser dissecting, we obtained a relatively small sample size of the TE cells. Enzyme digestion of the whole blastocyst combined with cell type identification followed by RNA-seq could help to obtain almost all cells in each embryo. The “whole blastocyst” genome analysis would reveal more information about the mosaicism characteristics of human embryos in the future.

CNVs of preimplantation embryos are a result of meiotic and/or mitotic errors, and mosaicism should be caused by mitotic errors. By tracking the origin of CNVs in the blastocysts, it was observed that meiotic errors do not contribute to mosaicism (S4 Fig). This could explain the observation that although oocyte aneuploidy increases with maternal age, the incidence of mosaicism is comparatively stable in different ages [26].

Using single cell sequencing, we observed “complementary” (deletion and duplication in the same chromosome region) CNVs in individual cells from the same embryo (Fig 4A). The complementary cells are likely daughter cells from one cell, with the aneuploidy brought by errors during mitosis. Assessment based on multi-cell biopsies may cover this aneuploidy in single cell (Fig 4B). These might cause false-negative diagnoses in clinical PGT-A.

In conclusion, single cell sequencing was applied to human blastocysts and hESCs, to reveal the incidence and characteristics of chromosome mosaicism in different cell lineages. In TE, the incidence of mosaicism was 65.71%, while ICM showed an incidence of 60.00%. There was no significant difference in the mosaic rates between TE and ICM. Tracking the origin of CNVs, we found that meiotic errors did not contribute to mosaicism. In addition, single cell sequencing could reveal more CNVs covered by multi-cell TE biopsies, which may help to explain false-negative diagnoses in PGT-A. Our study provided new insights into chromosome mosaicism and brought evidence that mosaicism could raise challenges in the clinical management of preimplantation embryo genetic analysis.

## Materials and methods

### Ethics statement

Human embryos were originally created for procreation, were PGT-A tested and diagnosed as aneuploid by fertility healthcare providers (independent of research described below) and were determined to be “not suitable for implantation” by fertility healthcare providers and parental embryo donors, as well as redundant euploid embryos after IVF treatment donated by parental embryo donors. This study has been reviewed and approved through the Embryo Research Oversight (EMRO) process performed by the Ethics Committee of Peking University Health Science Center (IRB00001052-16074). Written informed consent for embryo donation was obtained from the parental embryo donors as outlined by NIH guidelines, and de-identified embryo analysis and hESC line derivation was performed under the University of Michigan’s Institutional Review Board approved study (HUM00028742).

### Embryo biopsy and single-cell separation

All embryos used in this study were collected from the department of obstetrics and gynecology, University of Michigan. Donated blastocysts were cryopreserved individually by fertility healthcare providers (one embryo per cryopreservation device). Following warming and 6-24hrs growth of individual blastocysts, they were removed from the zona pellucida, and the ICM/polar TE was separated with the mural TE by micro-manipulation and laser-dissection using an inverted Hoffman modulation phase-contrast microscope. The mural TE was used to produce a single cell suspension. The ICM/polar TE was further dissected to obtain ICM cells, and the rest of ICM/polar TE cluster was plated onto mitotically-inactivated human foreskin fibroblasts as previously reported for hESC derivation [27,28]. After 3–4 days, the ICM/early epiblast was recognized as a small clump of cells. With micromanipulation, a small clump of the ICM was isolated to get single cells. The left ICM/early epiblast was allowed to expand/passage to produce hESCs as described below. Single cell suspensions were produced by gentle, repeated pipetting following treatment with Accutase/trypsin for 30 min at 37°C. Individual cells from each region were subsequently washed, microscopically-visually confirmed individualization, placed into a tube with 5μl lyses buffer (KT110700150, Yikon), snap frozen at -196°C, and stored at -80°C until processed for whole genome amplification (WGA) and DNA analysis.

### hESCs culture

hESCs were derived and cultured according to previous studies [27–29]. Briefly, the ICM was plated onto mitotically inactivated human foreskin fibroblast in hESC-Xeno-free Culture Media (Knock-out DMEM (Gibco, #10829) containing 20% Knockout Serum Replacement (Gibco, #12618012), 1mM Glutamax (Gibco, #35050–061), 0.1mM β-mercaptoethanol (Sigma-Aldrich, #M6250), 10mM Non-essential Amino Acid 100x (Gibco, #11140–05), 4 ng/ml basic Fibroblast Growth Factor-Xeno-free (MilliporeSigma, #GF003AF-100UG). Culture conditions for cell expansion on HFFs were maintained at 5% CO_2_/5% O_2_/90% N_2_ at 37°C, and hESCs were passage manually using silica glass microtools. Between passage 4–8 hESC lines were passage onto feeder cell-free matrix, Matrigel (Corning, #354277) with mTeSR1 Media (StemCell Technologies, #85850), with culture conditions of 5% CO_2_/20% O_2_/remainder air at 37°C. Early hESC freeze-backs were performed before passage 10. All pluripotency and embryoid body formation/linage marker characterization work were performed on hESCs expanded on Matrigel (S1 Fig). To get hESC single cells, undifferentiated hESCs clumps from different clones were collected off Matrigel during passage and digested with accutase/tryspin at 37°C for 1hr.

### WGA and NGS

WGA was performed with the multiple annealing and looping-based amplification cycles (MALBAC) kit (Yikon Genomics Inc.) according to the previously reported protocol [30]. Briefly, single cells were lysed by heating at 50°C for 3hr and 80°C for 10min. 30μl pre-Amp mix was added to the lysed product and incubated at 94°C for 3min. Then the product was amplified for 8 cycles of 20°C for 40sec, 30°C for 40sec, 40°C for 30sec, 50°C for 30sec, 60°C for 30sec, 70°C for 4 min, 95°C for 20sec, 58°C for 10sec, and pause at 4°C. 30μl amplification mix was added to the pre-Amp product and heated at 94°C for 30sec, and 17 cycles of 94°C for 20sec, 58°C for 30sec, and 3min at 72°C. The MALBAC product was purified and fragmented by Covaris into 300 bp. Sequencing libraries were constructed with the NEBNext Ultra DNA library Prep kit (New England Biolabs, Inc.). Illumina HiSeq 4000 platform was used for sequencing.

### Sequencing data processing

The sequencing reads were processed as previously described [31]. Briefly, the MALBAC amplification primers, Illumina sequencing adapters and low-quality bases were trimmed by in-house scripts. The processed reads were mapped to the human reference genome (hg38) using BWA-MEM algorithm with default settings [32]. The PCR duplications were removed and only uniquely mapped reads were retained for further analysis [33].

### Copy number analysis

The chromosome copy number of each single cell was estimated as the previously reported method [2] with the following minor modifications. First, the mapped reads were binned with a window of 1 Mb using readCounter software. Second, HMMcopy was used to detect the CNVs by controlling reference mappability and GC content [16].

The HMMcopy output corrected read counts were further used to evaluate the variability among samples. At first, the SDs of the corrected read counts within 30 sliding windows were computed for each chromosome, and then we would take the average SD for further analysis. The average SD of the five chromosomes that had the highest variability was defined as the variability score (VS) for every sample.

In this study, we carried out the following strategies to confirm the accuracy of the reported CNV. (1) Because the CNV determination is largely influenced by the amplification variability of single cell, we used a stringent criterion to exclude the cells with high amplification variability (top 15% cells with highest VS were discarded). (2) In CNV determination, a relatively larger bin can largely reduce the noise, therefore, we counted the reads in 1 Mb bin and only CNV larger than 10 Mb were reported, which guaranteed the accuracy of the reported CNV.

For each embryo, the mosaic rate of TE was defined as the number of TE cells with non-major karyotype divided by the total number of TE cells of this embryo. And the mosaic rates of ICM and hESC of each embryo were calculated with the same strategy.

### Statistical analyses

No statistical methods were used to pre-determine the sample size. The statistical analyses of mosaic rate in each figure were performed using R (version 3.4.1), and the P values were calculated by two-tailed unpaired Student’s t-test.

## Supporting information

S1 Fig Derivation and characterization of hESCs. A) Representative micrographs of day (D) 5 human blastocyst following thawing (21hr post-thaw); laser dissection of inner cell mass (ICM); plating of the ICM on human foreskin fibroblast feeder cells (HFFs) resulting in attachment (passage 0 –P0, day 2 –D2), and epiblast (EpiBl) outgrowth; and resultant derivation of hESC colonies (P1D6). Scale bar represents 100μm. B**)** Representative micrographs of hESCs stained by immunofluorescence to detect pluripotency markers NANOG, OCT3/4, SOX2, TRA-1-60, and SSEA4 (red) with Hoechst nuclear staining (blue), and brightfield (BF) images to assess morphology. All hESC lines used in this study were subjected to similar immunofluorescent staining. Scale bar ~50μm. C) RT-qPCR detection of the pluripotency markers *OCT3/4*, *NANOG*, and *SOX2*; including relative quantitation by RT-qPCR of *NANOG*, *OCT3/4*, and *SOX2* in relation to *β-Actin* (*ACTB*). D) Representative table/micrograph of hESC-derived embryoid bodies (EB, Day 7) determination of EB expression of differentiation markers. Relative quantitation by RT-qPCR of lineage markers in hESC-derived EBs normalized to *β-Actin*. RT-qPCR and gel detection of size appropriate amplicons representing lineage markers (*AFP*, *GATA-4* –two independent endoderm markers; *BRACHY*, *VE-CAD*–two independent mesoderm markers; and *TuJ-1*, *KRT-18 –*two independent ectoderm markers).
(TIF)

S2 Fig Quality analysis and exclusion of uninterpretable cells. A) Representative segmentation plots of two cells that were excluded from the analysis (top and upper-middle) and two cells that were included in the analysis (lower middle and bottom). Excluded cells had a wide spread of read counts, which showed high variability scores (VS). B) Box plots of VS for analyzed (n = 438) and excluded (n = 77) cells. n, number of cells. C) Density plot of VS for 438 analyzed cells. The 25th, 50th and 75th percentile of the VS were shown on the top right. D) Representative segmentation plots of three cells with the 25th, 50th and 75th percentile of the VS, respectively. E) Box plots of the VS for analyzed TE, ICM and hESC cells. F) Table summarizing the number of cells for TE (n = 285), ICM (n = 105) and hESC (n = 48) that were analyzed in this study. n, number of cells. G) Table summarizing the number of embryos that were analyzed in this study.
(TIF)

S3 Fig Mosaic rates versus sequenced cell numbers and distribution of aneuploidy of each chromosome in TE, ICM and hESC. A) Dot plot showing the mosaic rate versus sequenced cell numbers in the mosaic TE (n = 23), ICM (n = 9) and hESC (n = 2). n, number of embryos. B) Distribution of whole (top) and segmental (bottom) chromosome aneuploidy of each chromosome in TE, ICM and hESC.
(TIF)

S4 Fig Mosaic rates of meiotic and mitotic error embryos. A) Boxplot showing the mosaic rates of embryos with meiotic (n = 4), mitotic (n = 19) and meiotic&mitotic (n = 11) error. n, number of embryos.
(TIF)

S5 Fig Analysis of chromosome copy number of ICM and corresponding hESC from the same embryo. A) Chromosome status of the matched ICM-hESC in each embryo (n = 2). n, number of embryos. B) Mosaic rates of the matched ICM-hESC in each embryo.
(TIF)

S1 Table Summary of sequencing data coverage in each cell. (XLSX)

S2 Table Karyotype of embryos showed meiotic errors. seg: segmental aneuploid. (DOCX)

S3 Table Karyotype of embryos showed mitotic errors. seg: segmental aneuploid. (DOCX)

S4 Table Karyotype of embryos showed meiotic and mitotic errors. seg: segmental. (DOCX)

S5 Table Karyotype of euploid embryos. (DOCX)
