# Supplementary figures and images for: Regional and developmental characteristics of human embryo mosaicism revealed by single cell sequencing
Source: PLoS Genet. 2022 Aug 8;18(8):e1010310. doi: 10.1371/journal.pgen.1010310 (PMC9387924; doi:10.1371/journal.pgen.1010310)

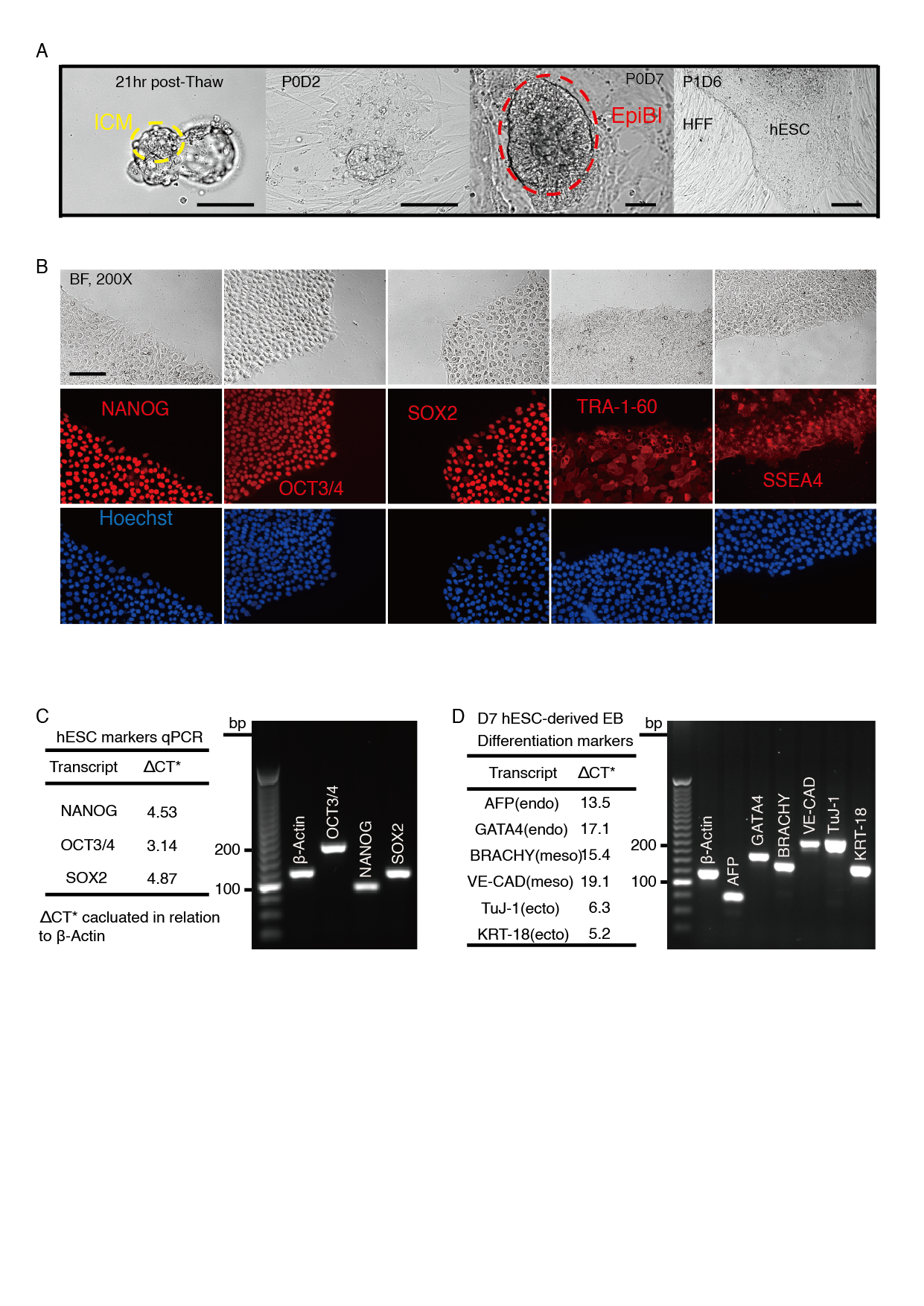

Supplement: S1 Fig — A) Representative micrographs of day (D) 5 human blastocyst following thawing (21hr post-thaw); laser dissection of inner cell mass (ICM); plating of the ICM on human foreskin fibroblast feeder cells (HFFs) resulting in attachment (passage 0 –P0, day 2 –D2), and epiblast (EpiBl) outgrowth; and resultant derivation of hESC colonies (P1D6). Scale bar represents 100μm. B) Representative micrographs of hESCs stained by immunofluorescence to detect pluripotency markers NANOG, OCT3/4, SOX2, TRA-1-60, and SSEA4 (red) with Hoechst nuclear staining (blue), and brightfield (BF) images to assess morphology. All hESC lines used in this study were subjected to similar immunofluorescent staining. Scale bar ~50μm. C) RT-qPCR detection of the pluripotency markers OCT3/4, NANOG, and SOX2; including relative quantitation by RT-qPCR of NANOG, OCT3/4, and SOX2 in relation to β-Actin (ACTB). D) Representative table/micrograph of hESC-derived embryoid bodies (EB, Day 7) determination of EB expression of differentiation markers. Relative quantitation by RT-qPCR of lineage markers in hESC-derived EBs normalized to β-Actin. RT-qPCR and gel detection of size appropriate amplicons representing lineage markers (AFP, GATA-4 –two independent endoderm markers; BRACHY, VE-CAD–two independent mesoderm markers; and TuJ-1, KRT-18 –two independent ectoderm markers). (TIF) [file pgen.1010310.s001.tif]

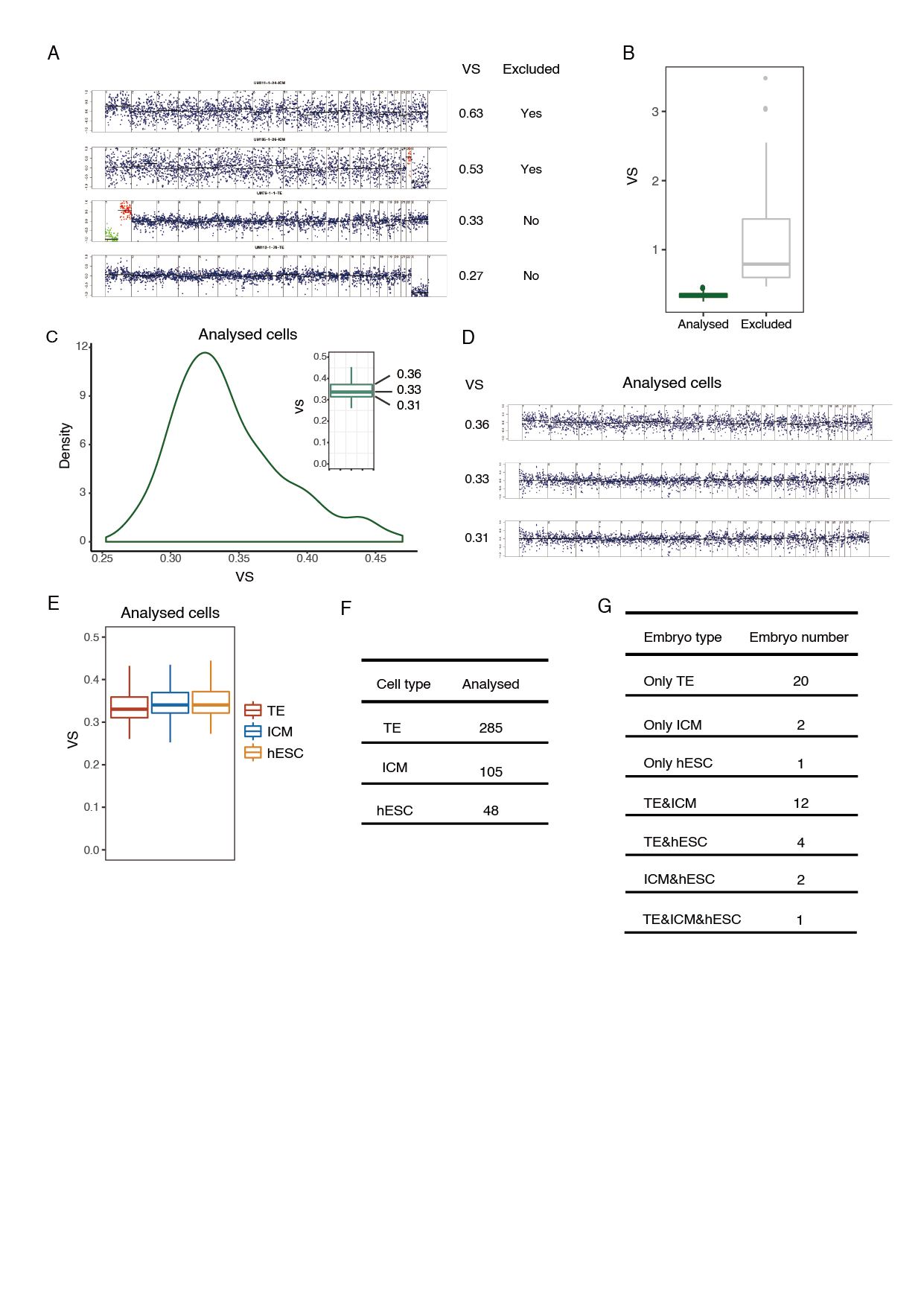

Supplement: S2 Fig — A) Representative segmentation plots of two cells that were excluded from the analysis (top and upper-middle) and two cells that were included in the analysis (lower middle and bottom). Excluded cells had a wide spread of read counts, which showed high variability scores (VS). B) Box plots of VS for analyzed (n = 438) and excluded (n = 77) cells. n, number of cells. C) Density plot of VS for 438 analyzed cells. The 25th, 50th and 75th percentile of the VS were shown on the top right. D) Representative segmentation plots of three cells with the 25th, 50th and 75th percentile of the VS, respectively. E) Box plots of the VS for analyzed TE, ICM and hESC cells. F) Table summarizing the number of cells for TE (n = 285), ICM (n = 105) and hESC (n = 48) that were analyzed in this study. n, number of cells. G) Table summarizing the number of embryos that were analyzed in this study. (TIF) [file pgen.1010310.s002.tif]

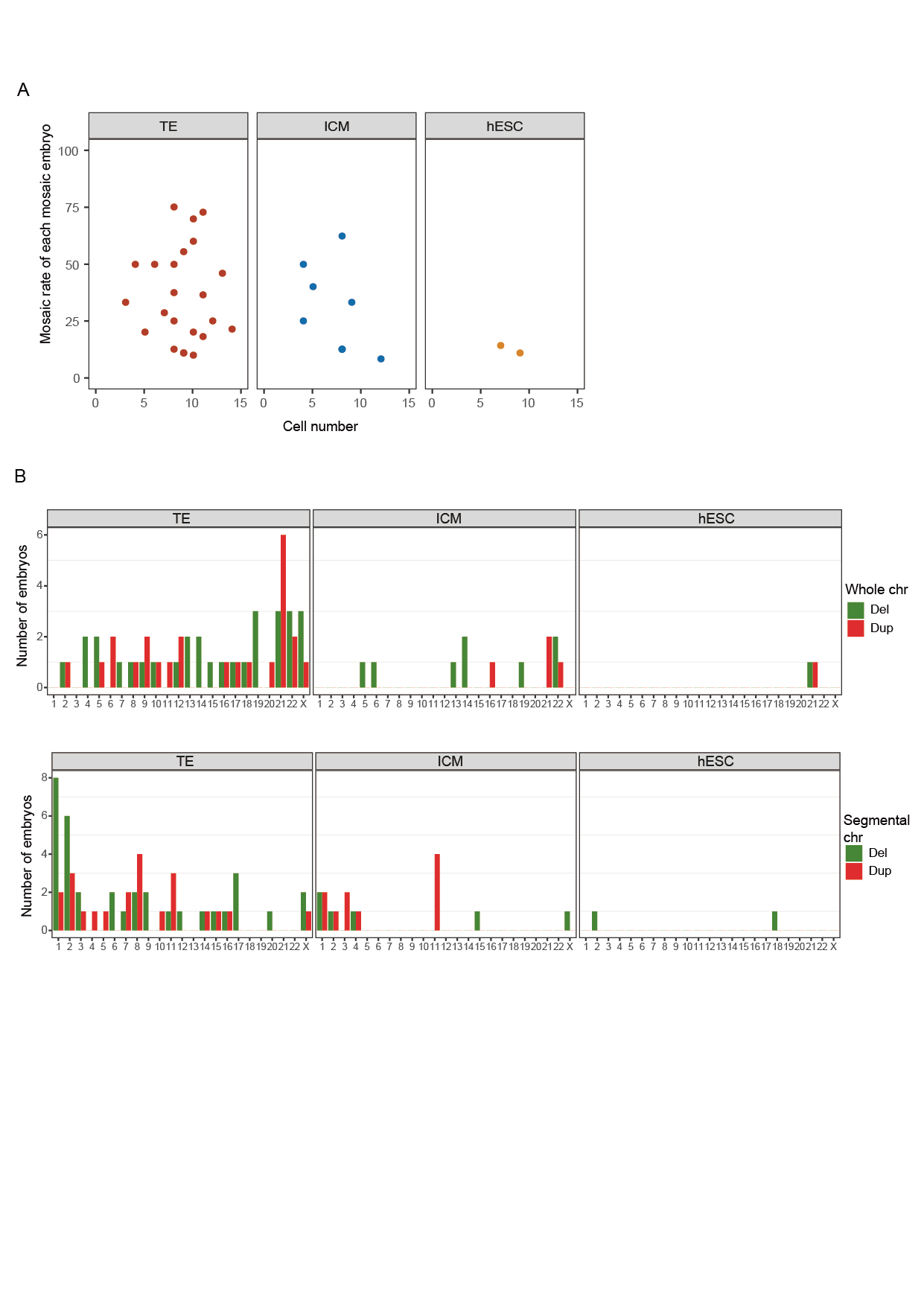

Supplement: S3 Fig — A) Dot plot showing the mosaic rate versus sequenced cell numbers in the mosaic TE (n = 23), ICM (n = 9) and hESC (n = 2). n, number of embryos. B) Distribution of whole (top) and segmental (bottom) chromosome aneuploidy of each chromosome in TE, ICM and hESC. (TIF) [file pgen.1010310.s003.tif]

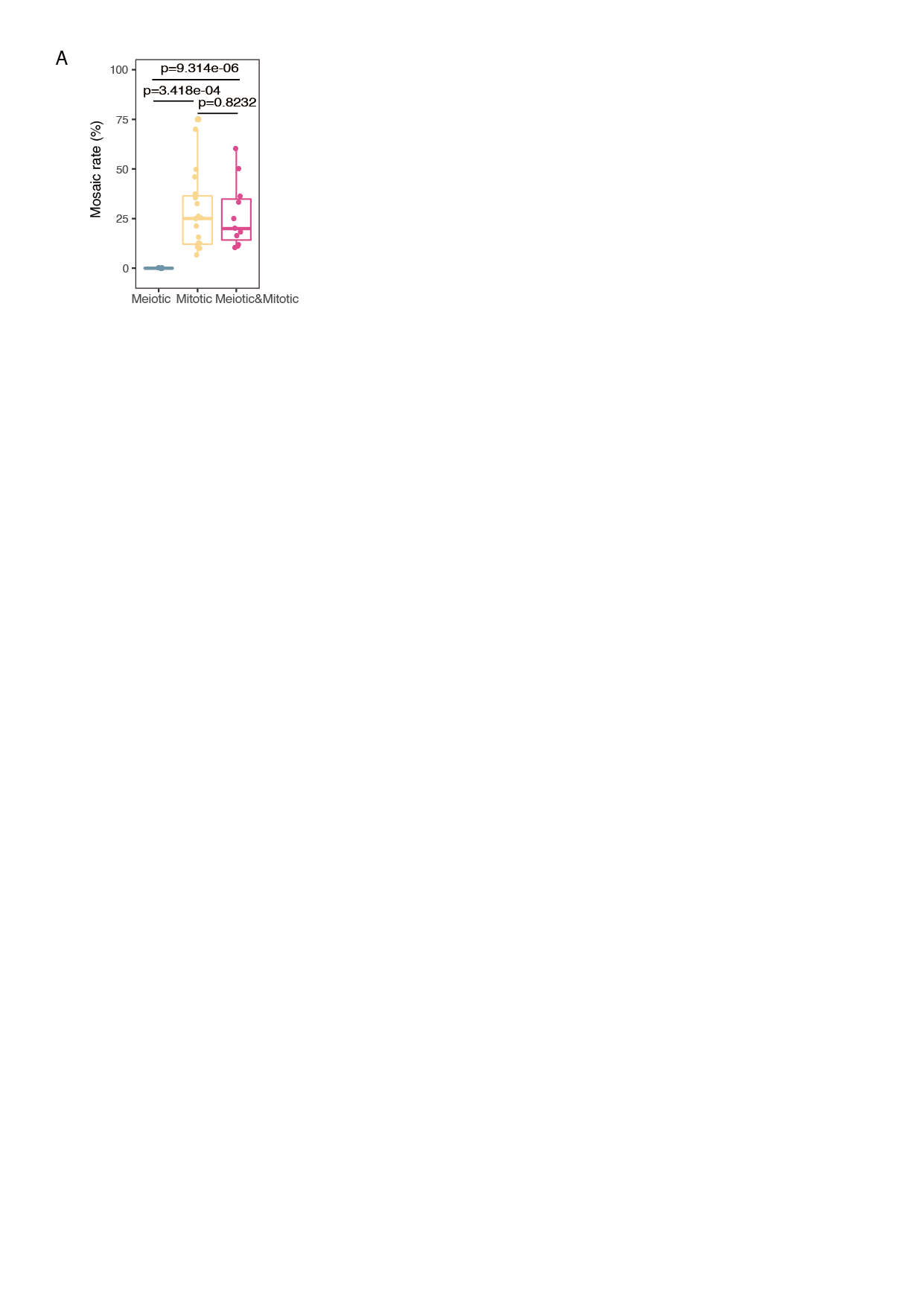

Supplement: S4 Fig — A) Boxplot showing the mosaic rates of embryos with meiotic (n = 4), mitotic (n = 19) and meiotic&mitotic (n = 11) error. n, number of embryos. (TIF) [file pgen.1010310.s004.tif]

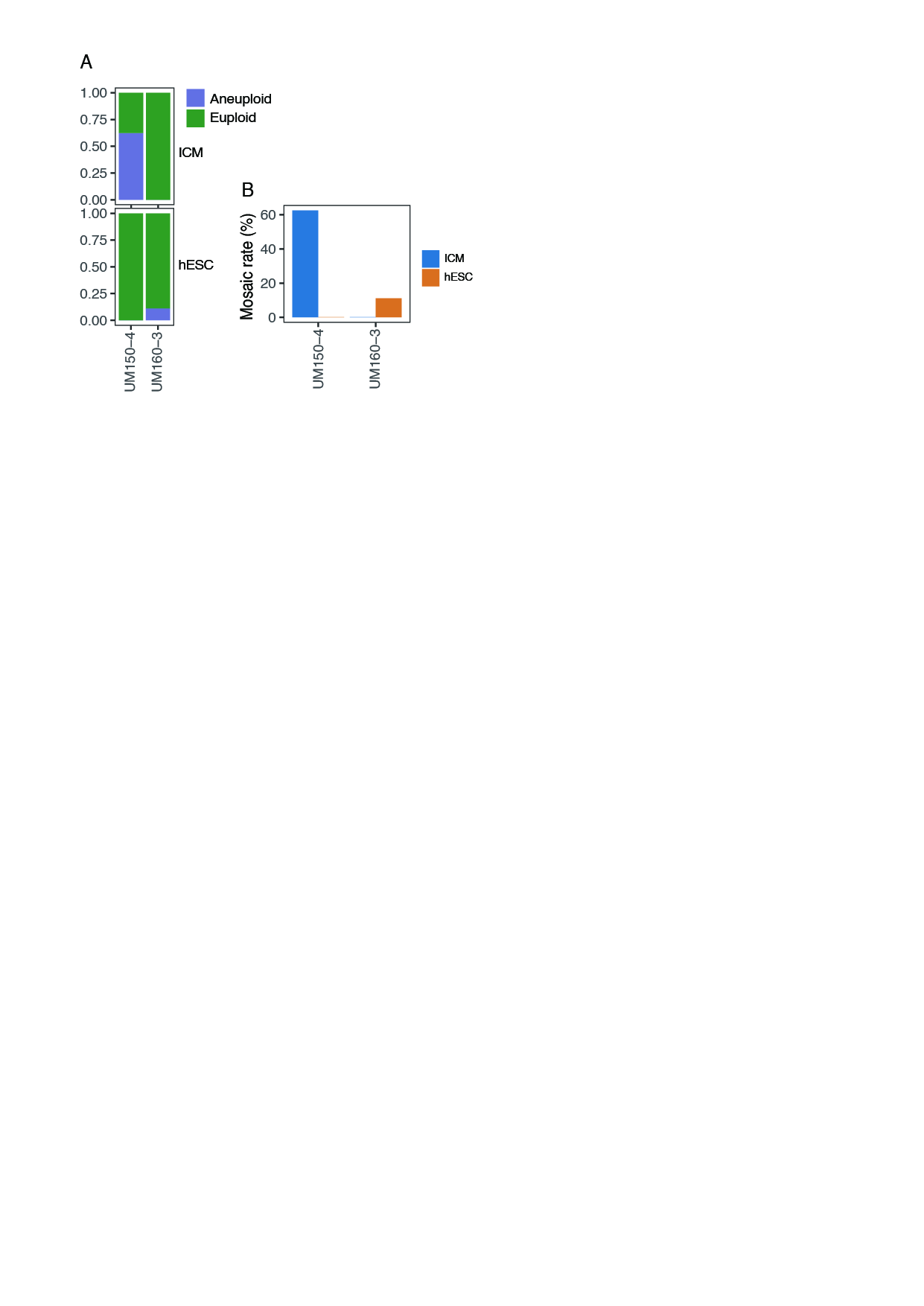

Supplement: S5 Fig — A) Chromosome status of the matched ICM-hESC in each embryo (n = 2). n, number of embryos. B) Mosaic rates of the matched ICM-hESC in each embryo. (TIF) [file pgen.1010310.s005.tif]
