# Supplementary material for: Regional and developmental characteristics of human embryo mosaicism revealed by single cell sequencing
Source: PLoS Genet. 2022 Aug 8;18(8):e1010310. doi: 10.1371/journal.pgen.1010310 (PMC9387924; doi:10.1371/journal.pgen.1010310)
Supplement: S2 Table — (DOCX) [file pgen.1010310.s007.docx]

**S2 Table: Karyotype of embryos showed meiotic errors in single cell sequencing results. seg: segmental aneuploid**

| **Embryoid** | **Age (wife)** | **Initial diagnostic results (multi-cell)** | | **Cellid** | **Type** | **Karyotype** |
| --- | --- | --- | --- | --- | --- | --- |
| UM151-3 | 30 | | 45,XX,-seg11,-16 | 56 | TE | 45,XX,-seg11,-16 |
|  |  |  |  | 57 | TE | 45,XX,-seg11,-16 |
|  |  |  |  | 58 | TE | 45,XX,-seg11,-16 |
| UM152-2 | 42 | | 45,XX,-19 | 21 | TE | 46,XX,-19,+22 |
|  |  |  |  | 22 | TE | 46,XX,-19,+22 |
|  |  |  |  | 23 | TE | 46,XX,-19,+22 |
|  |  |  |  | 24 | TE | 46,XX,-19,+22 |
|  |  |  |  | 25 | TE | 46,XX,-19,+22 |
|  |  |  |  | 26 | TE | 46,XX,-19,+22 |
|  |  |  |  | 28 | TE | 46,XX,-19,+22 |
|  |  |  |  | 29 | TE | 46,XX,-19,+22 |
|  |  |  |  | 30 | TE | 46,XX,-19,+22 |
|  |  |  |  | 31 | TE | 46,XX,-19,+22 |
|  |  |  |  | 33 | TE | 46,XX,-19,+22 |
| UM152-5 | 42 | | 47,XX,+21 | 2 | TE | 47,XX,+21 |
|  |  |  |  | 3 | TE | 47,XX,+21 |
|  |  |  |  | 4 | TE | 47,XX,+21 |
|  |  |  |  | 5 | TE | 47,XX,+21 |
|  |  |  |  | 6 | TE | 47,XX,+21 |
|  |  |  |  | 7 | TE | 47,XX,+21 |
|  |  |  |  | 8 | TE | 47,XX,+21 |
| UM189-1 | 31 | | 45,XY,-14 | 2 | TE | 45,XY,-14 |
|  |  |  |  | 3 | TE | 45,XY,-14 |
|  |  |  |  | 4 | TE | 45,XY,-14 |
|  |  |  |  | 5 | TE | 45,XY,-14 |
|  |  |  |  | 7 | TE | 45,XY,-14 |
|  |  |  |  | 8 | TE | 45,XY,-14 |
|  |  |  |  | 14 | TE | 45,XY,-14 |
|  |  |  |  | 15 | TE | 45,XY,-14 |
|  |  |  |  | 16 | TE | 45,XY,-14 |
|  |  |  |  | 17 | TE | 45,XY,-14 |
|  |  |  |  | 18 | TE | 45,XY,-14 |
|  |  |  |  | 19 | TE | 45,XY,-14 |
|  |  |  |  | 47 | ICM | 45,XY,-14 |
|  |  |  |  | 48 | ICM | 45,XY,-14 |
|  |  |  |  | 49 | ICM | 45,XY,-14 |
|  |  |  |  | 53 | ICM | 45,XY,-14 |
|  |  |  |  | 55 | ICM | 45,XY,-14 |
