# Supplementary material for: Regional and developmental characteristics of human embryo mosaicism revealed by single cell sequencing
Source: PLoS Genet. 2022 Aug 8;18(8):e1010310. doi: 10.1371/journal.pgen.1010310 (PMC9387924; doi:10.1371/journal.pgen.1010310)
Supplement: S3 Table — (DOCX) [file pgen.1010310.s008.docx]

**S3 Table: Karyotype of embryos showed mitotic errors in single cell sequencing results. seg: segmental aneuploid**

| **Embryoid** | **Age (wife)** | **Initial diagnostic results (multi-cell)** | **Cellid** | **Type** | **Karyotype** |
| --- | --- | --- | --- | --- | --- |
| UM4-9 | NA | NA | 1 | TE | 46,XY |
|  |  |  | 2 | TE | 46,XY |
|  |  |  | 3 | TE | 46,XY |
|  |  |  | 4 | TE | 46,XY |
|  |  |  | 5 | TE | 46,XY,+seg10 |
|  |  |  | 6 | TE | 46,XY,-seg1 |
|  |  |  | 7 | TE | 46,XY,+seg7 |
|  |  |  | 11 | TE | 46,XY |
|  |  |  | 12 | TE | 46,XY |
|  |  |  | 13 | TE | 46,XY |
|  |  |  | 14 | TE | 46,XY |
|  |  |  | 15 | TE | 46,XY |
|  |  |  | 17 | TE | 46,XY |
|  |  |  | 18 | TE | 46,XY |
| UM4-10 | NA | NA | 26 | TE | 46,XY |
|  |  |  | 27 | TE | 46,XY,-seg1,-seg3,-seg8 |
|  |  |  | 28 | TE | 46,XY |
|  |  |  | 29 | TE | 46,XY |
|  |  |  | 30 | TE | 46,XY |
|  |  |  | 38 | TE | 46,XY |
|  |  |  | 42 | TE | 46,XY,-seg9 |
|  |  |  | 45 | TE | 46,XY,-seg7 |
| UM76-1 | 33 | 45,X | 1 | TE | 46,XX,-seg1,+seg1 |
|  |  |  | 2 | TE | 44,XX,-13,-14 |
|  |  |  | 3 | TE | 46,XX |
|  |  |  | 4 | TE | 46,XX |
|  |  |  | 5 | TE | 46,XX |
|  |  |  | 6 | TE | 46,XX |
|  |  |  | 7 | TE | 46,XX |
|  |  |  | 8 | TE | 46,XX |
|  |  |  | 9 | TE | 46,XX |
|  |  |  | 15 | TE | 45,XX,-15 |
|  |  |  | 17 | TE | 46,XX |
|  |  |  | 18 | TE | 46,XX |
| UM112-1 | 32 | 46,XY | 58 | TE | 46,XY |
|  |  |  | 59 | TE | 46,XY |
|  |  |  | 60 | TE | 46,XY |
|  |  |  | 62 | TE | 46,XY |
|  |  |  | 63 | TE | 46,XY |
|  |  |  | 65 | TE | 46,XY |
|  |  |  | 66 | TE | 46,XY |
|  |  |  | 67 | TE | 46,XY |
|  |  |  | 69 | TE | 46,XY,+seg8 |
| UM112-3 | 32 | NA | 75 | TE | 46,XX,-seg6 |
|  |  |  | 76 | TE | 46,XX |
|  |  |  | 77 | TE | 46,XX,-segX |
|  |  |  | 78 | TE | 47,XX,+12 |
|  |  |  | 80 | TE | 45,XX,-12 |
|  |  |  | 81 | TE | 46,XX |
|  |  |  | 86 | TE | 46,XX,-seg6 |
|  |  |  | 87 | TE | 46,XX,-seg6,+seg8 |
| UM134-1 | 26 | 46,XY | 102 | TE | 46,XY |
|  |  |  | 103 | TE | 46,XY |
|  |  |  | 106 | TE | 46,XY |
|  |  |  | 107 | TE | 46,XY |
|  |  |  | 108 | TE | 46,XY |
|  |  |  | 109 | TE | 46,XY |
|  |  |  | 110 | TE | 46,XY |
|  |  |  | 112 | TE | 46,XY |
|  |  |  | 113 | TE | 46,XY |
|  |  |  | 114 | TE | 45,XY,-seg2,-10 |
| UM145-1 | 36 | 46,XY,-seg3 | 1 | TE | 46,XY |
|  |  |  | 4 | TE | 46,XY,-seg9 |
|  |  |  | 5 | TE | 46,XY |
|  |  |  | 6 | TE | 46,XY |
|  |  |  | 7 | TE | 46,XY,-seg2 |
|  |  |  | 8 | TE | 46,XY,-seg15,-seg17 |
| UM150-4 | 34 | NA | 1 | ICM | 46,XX,+seg4 |
|  |  |  | 1 | hESC | 46,XX |
|  |  |  | 2 | ICM | 46,XX,-seg2 |
|  |  |  | 3 | ICM | 46,XX,+seg1,-seg2 |
|  |  |  | 3 | hESC | 46,XX |
|  |  |  | 4 | ICM | 46,XX,-seg1,-seg2 |
|  |  |  | 6 | ICM | 46,XX,+seg2 |
|  |  |  | 6 | hESC | 46,XX |
|  |  |  | 7 | ICM | 46,XX |
|  |  |  | 8 | ICM | 46,XX |
|  |  |  | 8 | hESC | 46,XX |
|  |  |  | 10 | ICM | 46,XX |
|  |  |  | 15 | hESC | 46,XX |
|  |  |  | 20 | hESC | 46,XX |
|  |  |  | 21 | hESC | 46,XX |
|  |  |  | 22 | hESC | 46,XX |
|  |  |  | 23 | hESC | 46,XX |
|  |  |  | 25 | hESC | 46,XX |
|  |  |  | 27 | hESC | 46,XX |
| UM151-1 | 30 | 47,XXY | 32 | TE | 45,XXY,+X,-8,-19 |
|  |  |  | 33 | TE | 45,XY,-5 |
|  |  |  | 34 | TE | 46,XY |
|  |  |  | 36 | TE | 47,XXY,+X |
|  |  |  | 37 | TE | 46,XY,-seg1 |
|  |  |  | 38 | TE | 46,XY |
|  |  |  | 39 | TE | 47,XXY,+X |
|  |  |  | 40 | TE | 46,XY |
|  |  |  | 41 | TE | 46,XY,-seg1 |
|  |  |  | 42 | TE | 47,XXY,+X,-seg1 |
| UM152-1 | 42 | 49,XXX,+2,+16 | 1 | TE | 46,X,-X,+21 |
|  |  |  | 2 | TE | 47,XX,+21 |
|  |  |  | 4 | TE | 46,XX,-seg2,+seg4 |
|  |  |  | 5 | TE | 46,XX,-4,+21 |
|  |  |  | 6 | TE | 47,XX,+21 |
|  |  |  | 7 | TE | 47,XX,+21 |
|  |  |  | 9 | TE | 47,XX,+seg2,+21 |
|  |  |  | 11 | TE | 47,XX,+21 |
|  |  |  | 18 | TE | 48,XX,+17,+21 |
|  |  |  | 60 | ICM | 47,XX,+21 |
|  |  |  | 61 | ICM | 47,XX,+seg11,+21 |
|  |  |  | 63 | ICM | 47,XX,+21 |
|  |  |  | 64 | ICM | 47,XX,+21 |
|  |  |  | 65 | ICM | 47,XX,+21 |
|  |  |  | 66 | ICM | 47,XX,+21 |
|  |  |  | 67 | ICM | 47,XX,+21 |
|  |  |  | 68 | ICM | 47,XX,+21 |
| UM154-1 | 41 | 45,XX,-9 | 31 | TE | 46,XX |
|  |  |  | 32 | TE | 46,XX |
|  |  |  | 34 | TE | 46,XX |
|  |  |  | 36 | TE | 46,XX |
|  |  |  | 37 | TE | 44,XX,-13,-21 |
|  |  |  | 38 | TE | 46,XX |
|  |  |  | 39 | TE | 46,XX |
|  |  |  | 40 | TE | 46,XX |
| UM160-3 | 31 | 46,XX,+seg4 | 3 | TE | 46,XX,+seg3 |
|  |  |  | 5 | TE | 46,XX |
|  |  |  | 6 | TE | 46,XX,+seg1,-seg1,+seg2,+seg15 |
|  |  |  | 7 | TE | 46,XX,+segX,-seg8 |
|  |  |  | 12 | TE | 46,XX,+seg1,-seg12 |
|  |  |  | 13 | TE | 48,XX,+2,+8 |
|  |  |  | 14 | ICM | 46,XX |
|  |  |  | 14 | TE | 46,XX,-seg8 |
|  |  |  | 15 | ICM | 46,XX |
|  |  |  | 15 | TE | 46,XX |
|  |  |  | 17 | ICM | 46,XX |
|  |  |  | 18 | ICM | 46,XX |
|  |  |  | 19 | ICM | 46,XX |
|  |  |  | 20 | ICM | 46,XX |
|  |  |  | 22 | ICM | 46,XX |
|  |  |  | 23 | ICM | 46,XX |
|  |  |  | 25 | TE | 46,XX,+seg8 |
|  |  |  | 26 | TE | 46,XX |
|  |  |  | 27 | TE | 46,XX,+seg3 |
|  |  |  | 31 | hESC | 46,XX |
|  |  |  | 32 | hESC | 46,XX |
|  |  |  | 33 | hESC | 46,XX |
|  |  |  | 34 | hESC | 46,XX |
|  |  |  | 36 | hESC | 45,XX,-seg18,-21 |
|  |  |  | 40 | hESC | 46,XX |
|  |  |  | 42 | hESC | 46,XX |
|  |  |  | 43 | hESC | 46,XX |
|  |  |  | 44 | hESC | 46,XX |
| UM161-1 | 36 | 46,XX | 1 | TE | 46,XX |
|  |  |  | 3 | TE | 46,XX |
|  |  |  | 7 | TE | 46,XX |
|  |  |  | 8 | TE | 46,XX |
|  |  |  | 9 | TE | 46,XX |
|  |  |  | 10 | TE | 46,XX |
|  |  |  | 11 | TE | 46,XX |
|  |  |  | 12 | TE | 46,XX |
|  |  |  | 13 | TE | 46,XX |
|  |  |  | 21 | ICM | 46,XX |
|  |  |  | 22 | ICM | 46,XX |
|  |  |  | 25 | ICM | 46,XX,+seg3,+seg11 |
|  |  |  | 26 | ICM | 46,XX,-segX,-seg15 |
| UM162-1 | NA | NA | 62 | TE | 45,X,-X |
|  |  |  | 63 | TE | 45,X,-X |
|  |  |  | 64 | TE | 46,XX,-seg2 |
|  |  |  | 65 | TE | 45,X,-X |
|  |  |  | 67 | TE | 46,XX |
|  |  |  | 69 | TE | 46,XX,-seg17 |
|  |  |  | 70 | TE | 45,X,-X |
|  |  |  | 71 | TE | 46,XX |
|  |  |  | 73 | TE | 45,X,-X |
|  |  |  | 74 | TE | 46,XX |
|  |  |  | 75 | TE | 45,X,-X |
|  |  |  | 76 | TE | 45,X,-X |
|  |  |  | 77 | TE | 46,XX |
| UM163-2 | 33 | 47,XY,+9 | 23 | ICM | 46,XY,+seg11 |
|  |  |  | 24 | ICM | 46,XY |
|  |  |  | 25 | ICM | 46,XY |
|  |  |  | 26 | ICM | 46,XY |
|  |  |  | 27 | ICM | 46,XY |
|  |  |  | 28 | ICM | 46,XY |
|  |  |  | 29 | ICM | 46,XY |
|  |  |  | 30 | ICM | 46,XY |
| UM178-1 | 33 | 48,XX,+12,+22 | 1 | hESC | 46,XX |
|  |  |  | 1 | TE | 47,XX,+6 |
|  |  |  | 2 | hESC | 46,XX |
|  |  |  | 3 | hESC | 46,XX |
|  |  |  | 3 | TE | 45,XX,-9 |
|  |  |  | 4 | hESC | 46,XX |
|  |  |  | 5 | TE | 47,XX,+9 |
|  |  |  | 6 | hESC | 46,XX |
|  |  |  | 7 | TE | 46,XX |
|  |  |  | 9 | hESC | 46,XX |
|  |  |  | 9 | TE | 46,XX |
|  |  |  | 10 | hESC | 46,XX |
|  |  |  | 11 | hESC | 46,XX |
|  |  |  | 11 | TE | 46,XX,-seg1,+seg5 |
|  |  |  | 12 | TE | 46,XX |
|  |  |  | 13 | TE | 46,XX |
| UM191-1 | 31 | 47,XY,+22 | 4 | TE | 46,XY |
|  |  |  | 5 | TE | 46,XY |
|  |  |  | 8 | TE | 46,XY |
|  |  |  | 9 | TE | 46,XY |
|  |  |  | 10 | TE | 46,XY |
|  |  |  | 11 | TE | 46,XY |
|  |  |  | 12 | TE | 46,XY |
|  |  |  | 49 | ICM | 46,XY |
|  |  |  | 50 | ICM | 46,XY |
|  |  |  | 51 | ICM | 46,XY |
|  |  |  | 52 | ICM | 46,XY |
|  |  |  | 53 | ICM | 46,XY |
|  |  |  | 54 | ICM | 46,XY,+seg11 |
|  |  |  | 55 | ICM | 46,XY |
|  |  |  | 56 | ICM | 46,XY |
| UM205-1 | 29 | 46,XX,-seg20 | 1 | TE | 46,XX,-seg20 |
|  |  |  | 2 | hESC | 46,XX |
|  |  |  | 3 | TE | 46,XX |
|  |  |  | 5 | hESC | 46,XX |
|  |  |  | 5 | TE | 46,XX |
|  |  |  | 6 | TE | 46,XX |
|  |  |  | 8 | TE | 46,XX |
|  |  |  | 9 | TE | 46,XX |
|  |  |  | 10 | hESC | 46,XX |
|  |  |  | 10 | TE | 46,XX |
|  |  |  | 11 | TE | 45,XX,-22 |
|  |  |  | 12 | hESC | 46,XX |
|  |  |  | 14 | hESC | 46,XX |
|  |  |  | 15 | hESC | 46,XX |
|  |  |  | 18 | TE | 46,XX |
|  |  |  | 19 | TE | 46,XX |
|  |  |  | 20 | TE | 46,XX |
| UM211-1 | 35 | 45,XX,-17 | 1 | TE | 46,XX |
|  |  |  | 2 | TE | 46,XX |
|  |  |  | 6 | TE | 46,XX |
|  |  |  | 7 | TE | 45,XX,-17 |
|  |  |  | 8 | TE | 46,XX |
|  |  |  | 9 | TE | 46,XX |
|  |  |  | 10 | TE | 45,XX,-17 |
|  |  |  | 11 | TE | 46,XX |
|  |  |  | 12 | TE | 46,XX |
|  |  |  | 13 | TE | 46,XX |
|  |  |  | 23 | ICM | 46,XX |
|  |  |  | 25 | ICM | 46,XX |
|  |  |  | 26 | ICM | 46,XX |
|  |  |  | 27 | ICM | 46,XX |
|  |  |  | 29 | ICM | 46,XX |
|  |  |  | 31 | ICM | 46,XX |
|  |  |  | 34 | ICM | 46,XX |
|  |  |  | 35 | ICM | 46,XX |
|  |  |  | 36 | ICM | 46,XX |
