# Supplementary material for: Regional and developmental characteristics of human embryo mosaicism revealed by single cell sequencing
Source: PLoS Genet. 2022 Aug 8;18(8):e1010310. doi: 10.1371/journal.pgen.1010310 (PMC9387924; doi:10.1371/journal.pgen.1010310)
Supplement: S4 Table — (DOCX) [file pgen.1010310.s009.docx]

**S4 Table: Karyotype of embryos showed meiotic and mitotic errors in single cell sequencing results. seg: segmental aneuploid**

| **Embryoid** | **Age (wife)** | **Initial diagnostic results (multi-cell)** | **Cellid** | **Type** | **Karyotype** |
| --- | --- | --- | --- | --- | --- |
| UM146-1 | 41 | 48,XX,+12,+18 | 28 | TE | 48,XX,-seg2,-seg6,+12,+18 |
|  |  |  | 29 | TE | 48,XX,+12,+18 |
|  |  |  | 30 | TE | 48,XX,+12,+18 |
|  |  |  | 31 | TE | 49,XX,+6,+12,+18 |
|  |  |  | 32 | TE | 48,XX,+12,+18 |
|  |  |  | 34 | TE | 48,XX,+12,+18 |
|  |  |  | 35 | TE | 48,XX,+12,+18 |
|  |  |  | 36 | TE | 48,XX,+12,+18 |
|  |  |  | 38 | TE | 48,XX,+12,+18 |
|  |  |  | 39 | TE | 49,XX,+6,+12,+18 |
|  |  |  | 44 | TE | 49,XX,+6,+12,+18 |
| UM151-2 | 30 | 46,XX,+seg11 | 18 | TE | 46,XX,+seg11 |
|  |  |  | 19 | TE | 46,XX,+seg11 |
|  |  |  | 20 | TE | 46,XX,+seg11 |
|  |  |  | 22 | TE | 46,XX,+seg11 |
|  |  |  | 26 | TE | 46,XX,+seg11 |
|  |  |  | 48 | TE | 46,XX,+seg11 |
|  |  |  | 50 | TE | 45,XX,+seg11,-seg17,-18 |
|  |  |  | 51 | TE | 46,XX,+seg11 |
|  |  |  | 52 | TE | 46,XX,+seg11 |
| UM155-1 | NA | NA | 1 | TE | 45,XX,-5 |
|  |  |  | 3 | TE | 45,XX,-5 |
|  |  |  | 4 | TE | 44,XX,-seg1,-5,-21 |
|  |  |  | 10 | TE | 45,XX,-5 |
|  |  |  | 15 | TE | 45,XX,-5 |
|  |  |  | 29 | ICM | 45,XX,-5 |
|  |  |  | 30 | ICM | 45,XX,+seg1,-5 |
|  |  |  | 34 | ICM | 45,XX,-5 |
|  |  |  | 36 | ICM | 45,XX,-5 |
|  |  |  | 37 | ICM | 45,XX,-5 |
|  |  |  | 40 | ICM | 45,XX,-5 |
|  |  |  | 41 | ICM | 45,XX,-5 |
|  |  |  | 44 | ICM | 45,XX,-5 |
|  |  |  | 45 | ICM | 45,XX,-5 |
|  |  |  | 46 | ICM | 45,XX,-5 |
|  |  |  | 47 | ICM | 45,XX,-5 |
|  |  |  | 48 | ICM | 45,XX,-5 |
| UM156-2 | 34 | 45,X | 4 | TE | 45,X, |
|  |  |  | 6 | TE | 45,X, |
|  |  |  | 16 | TE | 43,X,-4,-7 |
| UM160-2 | 31 | NA | 33 | TE | 45,XX,+seg7,-21 |
|  |  |  | 35 | TE | 45,XX,-seg2,+seg8,-21 |
|  |  |  | 46 | TE | 45,XX,-21 |
|  |  |  | 52 | TE | 45,XX,-21 |
|  |  |  | 54 | TE | 45,XX,-21 |
|  |  |  | 55 | TE | 45,XX,+seg2,+seg8,-21 |
|  |  |  | 57 | TE | 45,XX,-seg14,-21 |
|  |  |  | 58 | TE | 45,XX,-segX,-seg3,+seg8,+seg14,-seg16,-21 |
|  |  |  | 59 | TE | 45,XX,-21 |
|  |  |  | 60 | TE | 46,XX,+20,-21 |
| UM177-1 | 35 | 45,XY,-22 | 1 | TE | 45,XY,-22 |
|  |  |  | 2 | TE | 45,XY,-22 |
|  |  |  | 3 | TE | 45,XY,-22 |
|  |  |  | 4 | TE | 45,XY,+seg11,-22 |
|  |  |  | 8 | TE | 46,XY,+5,-22 |
|  |  |  | 9 | TE | 45,XY,-22 |
|  |  |  | 10 | TE | 45,XY,-22 |
|  |  |  | 11 | TE | 45,XY,-22 |
|  |  |  | 19 | ICM | 43,XY,-13,-14,-22 |
|  |  |  | 21 | ICM | 45,XY,-22 |
|  |  |  | 22 | ICM | 45,XY,-22 |
|  |  |  | 23 | ICM | 45,XY,-22 |
| UM195-1 | 32 | 47,XY,+22 | 2 | TE | 47,XY,+22 |
|  |  |  | 4 | TE | 47,XY,+22 |
|  |  |  | 11 | TE | 47,XY,+22 |
|  |  |  | 12 | TE | 47,XY,+22 |
|  |  |  | 13 | TE | 47,XY,+22 |
|  |  |  | 18 | TE | 47,XY,+22 |
|  |  |  | 20 | ICM | 47,XY,+22 |
|  |  |  | 21 | ICM | 47,XY,+22 |
|  |  |  | 30 | ICM | 47,XY,+22 |
|  |  |  | 36 | ICM | 47,XY,-seg1,+22 |
|  |  |  | 38 | ICM | 47,XY,-seg4,+22 |
| UM196-1 | 38 | 46,XY,+16,-19 | 1 | TE | 46,XY,-seg1,+16,-19 |
|  |  |  | 3 | TE | 46,XY,+9,+seg16,-19 |
|  |  |  | 7 | TE | 46,XY,+16,-19 |
|  |  |  | 8 | TE | 46,XY,+16,-19 |
| UM201-1 | 40 | 45,XX,-19 | 3 | TE | 45,XX,-22 |
|  |  |  | 4 | TE | 45,XX,+seg11,-22 |
|  |  |  | 5 | TE | 45,XX,-22 |
|  |  |  | 7 | TE | 45,XX,-22 |
|  |  |  | 8 | TE | 44,XX,-2,-22 |
|  |  |  | 9 | TE | 45,XX,-22 |
|  |  |  | 10 | TE | 45,XX,-22 |
|  |  |  | 24 | ICM | 45,XX,-22 |
|  |  |  | 25 | ICM | 45,XX,-22 |
|  |  |  | 30 | ICM | 45,XX,-22 |
|  |  |  | 33 | ICM | 45,XX,-22 |
|  |  |  | 37 | ICM | 45,XX,-22 |
| UM202-1 | 34 | 47,XX,+21 | 2 | TE | 47,XX,+21 |
|  |  |  | 3 | TE | 47,XX,+21 |
|  |  |  | 7 | hESC | 47,XX,+21 |
|  |  |  | 8 | hESC | 46,XX,+21 |
|  |  |  | 8 | TE | 47,XX,+21 |
|  |  |  | 10 | hESC | 47,XX,-seg2,+21 |
|  |  |  | 11 | hESC | 47,XX,+21 |
|  |  |  | 12 | hESC | 47,XX,+21 |
|  |  |  | 16 | hESC | 47,XX,+21 |
|  |  |  | 17 | hESC | 47,XX,+21 |
| UM209-1 | 30 | 47,XY,+21 | 1 | TE | 47,XY,+21 |
|  |  |  | 2 | TE | 47,XY,+21 |
|  |  |  | 4 | TE | 47,XY,+21 |
|  |  |  | 7 | TE | 47,XY,+21 |
|  |  |  | 10 | TE | 47,XY,+21 |
|  |  |  | 14 | TE | 47,XY,+21 |
|  |  |  | 22 | ICM | 47,XY,+seg3,+21 |
|  |  |  | 23 | ICM | 47,XY,+21 |
|  |  |  | 24 | ICM | 47,XY,+21 |
|  |  |  | 27 | ICM | 47,XY,+21 |
|  |  |  | 28 | ICM | 46,XY,-6,+21 |
|  |  |  | 30 | ICM | 47,XY,+21 |
|  |  |  | 36 | ICM | 47,XY,+21 |
|  |  |  | 37 | ICM | 47,XY,+21 |
|  |  |  | 40 | ICM | 46,XY,-6,+21 |
