# Supplementary material for: Regional and developmental characteristics of human embryo mosaicism revealed by single cell sequencing
Source: PLoS Genet. 2022 Aug 8;18(8):e1010310. doi: 10.1371/journal.pgen.1010310 (PMC9387924; doi:10.1371/journal.pgen.1010310)
Supplement: S5 Table — (DOCX) [file pgen.1010310.s010.docx]

**S5 Table: Karyotype of embryos showed euploid in single cell sequencing results.**

| **Embryoid** | **Age (wife)** | **Initial diagnostic results (multi-cell)** | | **Cellid** | **Type** | **Karyotype** |
| --- | --- | --- | --- | --- | --- | --- |
| UM90-12 | 24 | | NA | 44 | hESC | 46,XX |
|  |  |  |  | 45 | hESC | 46,XX |
|  |  |  |  | 46 | hESC | 46,XX |
|  |  |  |  | 47 | hESC | 46,XX |
|  |  |  |  | 48 | hESC | 46,XX |
|  |  |  |  | 49 | hESC | 46,XX |
|  |  |  |  | 50 | hESC | 46,XX |
| UM139-2 | 32 | | 46,XY | 51 | TE | 46,XY |
|  |  |  |  | 53 | TE | 46,XY |
|  |  |  |  | 54 | TE | 46,XY |
| UM159-1 | 33 | | 46,XY | 61 | ICM | 46,XY |
|  |  |  |  | 63 | ICM | 46,XY |
|  |  |  |  | 65 | ICM | 46,XY |
|  |  |  |  | 66 | ICM | 46,XY |
|  |  |  |  | 67 | ICM | 46,XY |
| UM167-1 | 34 | | 47,XXY | 3 | TE | 46,XX |
|  |  |  |  | 4 | TE | 46,XX |
|  |  |  |  | 9 | TE | 46,XX |
|  |  |  |  | 10 | TE | 46,XX |
|  |  |  |  | 11 | TE | 46,XX |
|  |  |  |  | 18 | TE | 46,XX |
|  |  |  |  | 20 | TE | 46,XX |
|  |  |  |  | 21 | TE | 46,XX |
| UM191-2 | 31 | | 46,XX,+seg9 | 1 | TE | 46,XX |
|  |  |  |  | 2 | TE | 46,XX |
|  |  |  |  | 4 | TE | 46,XX |
|  |  |  |  | 6 | TE | 46,XX |
|  |  |  |  | 7 | TE | 46,XX |
|  |  |  |  | 8 | TE | 46,XX |
|  |  |  |  | 35 | ICM | 46,XX |
|  |  |  |  | 39 | ICM | 46,XX |
|  |  |  |  | 40 | ICM | 46,XX |
|  |  |  |  | 42 | ICM | 46,XX |
|  |  |  |  | 50 | ICM | 46,XX |
|  |  |  |  | 51 | ICM | 46,XX |
|  |  |  |  | 52 | ICM | 46,XX |
